# Body composition’s effect on the bone–vascular axis of osteoporosis discovered in AI-based CT analysis of COPD patients

**DOI:** 10.1007/s00330-026-12677-3

**Published:** 2026-07-03

**Authors:** Bettina K. Budai, Andreas Wagner, Ondrej Havlicek, Philip Konietzke, Franziska C. Trudzinski, Jürgen Biederer, Claus P. Heußel, Hans-Ulrich Kauczor, Viktoria Palm

**Affiliations:** 1 Department of Diagnostic and Interventional Radiology (DIR), Heidelberg University Hospital, Heidelberg, Germany; 2 Translational Lung Research Center (TLRC), German Center for Lung Research (DZL), University of Heidelberg, Heidelberg, Germany; 3 Diagnostic and Interventional Radiology with Nuclear Medicine, Thoraxklinik Heidelberg, Heidelberg, Germany; 4 Department of Diagnostic Radiology and Neuroradiology, University Medicine Greifswald, Greifswald, Germany; 5 Clinic for Nuclear Medicine, University Medicine Greifswald, Greifswald, Germany; 6 Department of Pneumology and Critical Care, Thoraxklinik Heidelberg gGmbH, Heidelberg, Germany; 7 Faculty of Medicine and Life Sciences, University of Latvia, Riga, Latvia; 8 Faculty of Medicine, Christian-Albrechts-Universität zu Kiel, Kiel, Germany

**Keywords:** Chronic obstructive pulmonary disease, Comorbidity, Vascular calcification, Artificial intelligence, Body composition

## Abstract

**Objectives:**

This study aimed to investigate the effect of body composition on the inverse relationship between vertebral bone density (T12 BMD) and total thoracic vascular calcification (TTVC) in patients with chronic obstructive pulmonary disease (COPD). Moreover, we aimed to assess whether intermuscular adipose tissue (IMAT) affects the bone-vascular axis.

**Materials and methods:**

Chest CT scans of 539 COPD patients from the multicentric prospective COSYCONET study were retrospectively analyzed using AI-based tools for T12 BMD, TTVC, and volumetric body composition. Multivariable linear regression models were built to investigate the effect of conventional body phenotypes (normal, sarcopenic, non-sarcopenic obesity, and sarcopenic obesity). Stepwise interaction model building included T12 BMD, IMAT, their interaction, adding BMI, clinical and metabolic covariates, lung function, physical performance, and age.

**Results:**

The T12 BMD showed a consistent inverse association with TTVC in all phenotypes, with β = −0.38 (*p* < 0.01) in normal nutritional status, β = −0.36 (*p* < 0.01) in sarcopenia, and β = −0.24 (*p* < 0.01) in non-sarcopenic obesity. However, the phenotype’s significant effect was not confirmed in the interaction model. Age and pack-years were associated with calcification, but IMAT remained independently associated (β = 0.15, 95% CI 0.015–0.28, *p* = 0.029), while the interaction between T12 BMD and IMAT lost significance once age was included.

**Conclusion:**

IMAT index was independently associated with TTVC in COPD. The modifying effect of IMAT on the bone-vascular axis was most evident in models without age adjustment, suggesting that the observed interaction may be influenced by age.

**Key Points:**

***Question*** *The interactions between body composition, sarcopenia, arteriosclerosis, and osteoporosis are not fully understood. AI-based CT analysis provides a more holistic picture of multimorbidity in COPD.*

***Findings*** *Intermuscular adipose tissue (IMAT) was independently associated with vascular calcification in stepwise-adjusted models. The interaction of IMAT and bone density is demonstrated but showed age-dependence.*

***Clinical relevance*** *Increased IMAT can capture a vulnerable COPD patient group with metabolic dysregulation and physical frailty, besides vascular aging. Muscle fat infiltration may indicate impaired musculoskeletal health and serve as a marker of arteriosclerosis beyond possible effects of age or BMI.*

**Graphical Abstract:**

**Figure:**
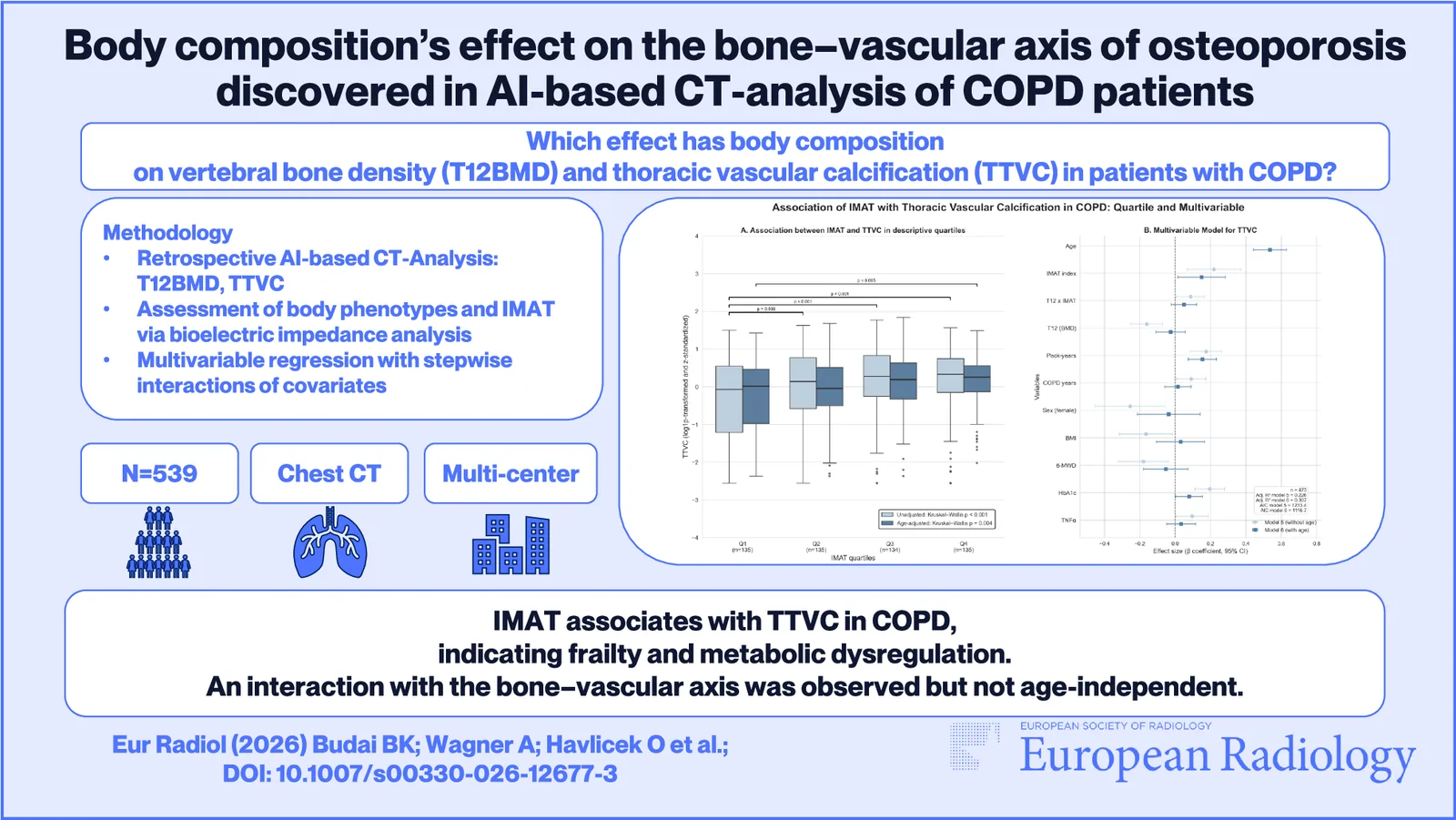

## Introduction

Chronic obstructive pulmonary disease (COPD) is a heterogeneous disease characterized by varying contributions of emphysema and airway abnormalities to lung function impairment, affecting approximately 384 million individuals, with a global prevalence of 11.7%, and is recognized as the third leading cause of death [1, 2]. Despite its widespread impact, traditional classification systems such as the Global Initiative for Chronic Obstructive Lung Disease (GOLD) staging are limited in their ability to reflect the clinical heterogeneity of COPD, particularly in terms of complex dynamic interactions with comorbidities [3–5].

In recent years, there has been increasing recognition of the need for personalized approaches to COPD management [6–8]. Variability in disease progression, symptom burden, and therapeutic response underscores the limitations of classical treatment paradigms [6, 9–11]. One area gaining attention is the role of body composition in influencing disease phenotype and prognosis. Recent studies indicate that over 50% of COPD patients with a normal BMI have a reduced fat‑free mass index [12]. Moreover, it is estimated that sarcopenia affects about 25–30% of COPD patients overall [13–17]. Altered body composition in COPD reflects systemic processes such as inflammation, metabolic dysfunction, and hormonal changes [14, 15, 18, 19]. These changes are associated with common COPD comorbidities, including osteoporosis and coronary artery disease [20–23]. Meta‑analyses report an osteoporosis prevalence of about 38% in COPD patients with vertebral fractures affecting more than 20% [24–27]. Similarly, coronary artery disease is highly prevalent (20–60%) in COPD and is influenced by visceral adiposity and muscle wasting [28–30]. These overlapping features suggest a shared pathophysiological basis that has yet to be assessed.

Despite these associations, the interplay between imaging-derived body composition phenotypes and comorbidities in COPD remains insufficiently characterized. Hence, the current study explores the associations of osteoporosis and arterial calcifications in different body composition phenotypes in a COPD cohort. With artificial intelligence (AI)-based analysis of CT scans, we aimed to explore the effect of body composition on the bone-vascular axis to guide future phenotype-specific, personalized strategies in risk stratification and management of COPD patients.

## Materials and methods

### Study population

**Fig. 1:**
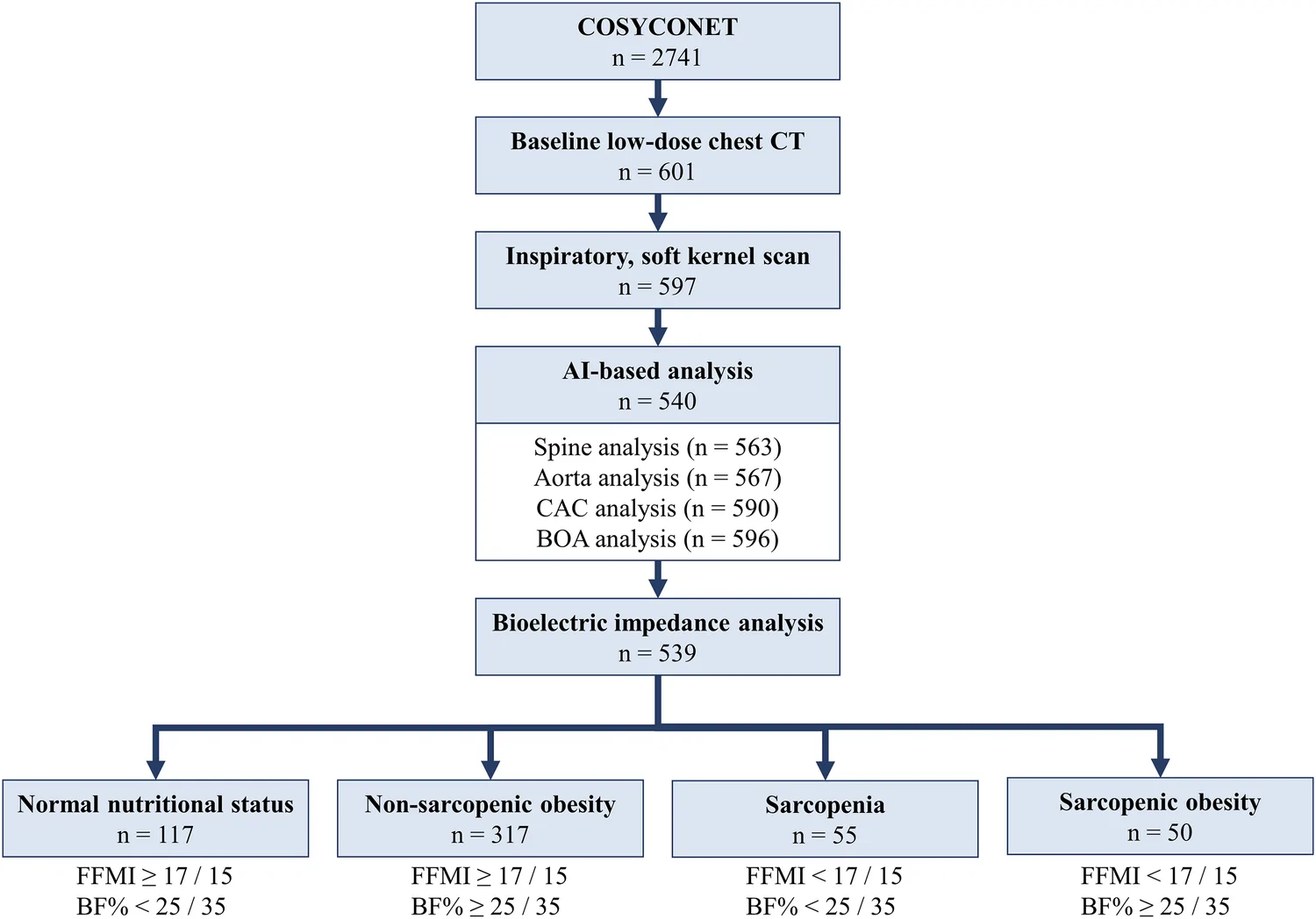
Flowchart of participant inclusion criteria using AI-based automated spine, vascular, and body composition analysis on the CT scans, as well as bioelectric impedance analysis for body composition phenotyping. Participants were categorized into four body phenotype groups according to sex-specific cutoff values: normal nutritional status, non-sarcopenic obesity, sarcopenic obesity, and sarcopenia. AI, artificial intelligence; BF, body fat; BOA, Body and Organ Analysis tool; CAC, coronary artery calcification; CT, computed tomography; FFMI, fat-free mass index

All study procedures received approval from the Ethics Committee of Philipps University Marburg (reference no. AZ 2010-28) and followed the ethical standards set by the revised Declaration of Helsinki. Prior to enrollment, written informed consent was obtained from all participants. The COSYCONET study is registered on ClinicalTrials.gov (ID: NCT01245933), and detailed methodology has been documented in previous publications [31]. A subgroup of 601 participants underwent low-dose computed tomography (LDCT). For this study, automated, AI-driven analyses were successfully performed for 540 patients. After quality control, one patient had to be excluded due to missing data on bioelectrical impedance analysis (BIA) used for the conventional body composition phenotyping. Thus, the final cohort consisted of 539 patients (Fig. 1).

### Data collection

#### Clinical and functional data

Demographic, clinical, and functional characteristics were obtained at the study visit closest to each participant’s CT scan. If data from that visit were missing, values from the next available follow-up were used to ensure completeness. Demographic and anthropometric data used in this study included age, sex, weight, body mass index (BMI), and anamnestic data, including the duration of COPD since diagnosis (in years), smoking (pack-years), and current use of oral or inhaled corticosteroids (ICS). Metabolic and inflammatory markers investigated included IL-6, IL-8, TNF, HbA1C, and HDL. Pulmonary function was evaluated using spirometry and diffusion capacity testing, with all reference values derived from the Global Lung Function Initiative standards. The primary pulmonary variables analyzed were forced expiratory volume in 1 s (FEV₁), forced vital capacity (FVC), Tiffeneau index, and diffusion capacity for carbon monoxide (DLCO). The Tiffeneau index (FEV₁/FVC) was used as an indicator of airflow limitation, representing the fraction of the forced vital capacity (FVC) exhaled during the first second of forced expiration (FEV₁) and reflecting expiratory airflow relative to lung volume. COPD severity was classified according to GOLD criteria, both by airflow limitation (GOLD 1–4) and by symptom and exacerbation burden (A–D). Physical activity and mobility evaluation included the Timed Up and Go (TUG) test, the 6-min walk distance (6-MWD), gait speed, and the International Physical Activity Questionnaire (IPAQ). Further clinical assessments included the BODE index, the COPD assessment test (CAT), and the modified Medical Research Council (mMRC) dyspnea scale.

#### Body composition phenotypes

Conventional body composition phenotypes were derived according to fat-free mass index (FFMI) and body fat percentage (BF%), applying sex-specific cutoffs. Individuals were classified as having normal nutritional status if FFMI was ≥ 17 kg/m² for men or ≥ 15 kg/m² for women [32] and BF% was < 25% for men or < 35% for women [33]. Non-sarcopenic obesity was defined by preserved FFMI in combination with elevated BF%. Sarcopenic obesity was assigned if FFMI was below the sex-specific threshold and BF% exceeded the defined cutoff. Sarcopenia was defined by both low FFMI and low BF%.

#### CT acquisition

Chest LDCT examinations were performed at multiple clinical sites across Germany, using a diverse range of scanner models from leading manufacturers. Equipment included systems by Siemens Healthineers, such as the SOMATOM Definition series, SOMATOM Force, and Sensation 40/64; GE Healthcare with models like Optima CT660 and LightSpeed VCT; and Philips Healthcare with the iCT 256. Despite the variability in hardware, the examination followed a standardized image acquisition using a non-contrast-enhanced, non-gated, low-dose protocol across sites. Imaging was conducted in full inspiration. The majority of scans employed a tube voltage of 120 kVp, though some were acquired at 100 or 140 kVp. For this study, thin-slice (typically 0.625 mm to 1.25 mm) axial scans were selected. The median time interval between the CT and the corresponding clinical visit was 50 days, with a standard deviation of 148.09 days.

#### AI-based chest CT analysis

The AI-Rad Companion Research Chest CT Explore V1.3 (AIRC) (Siemens Healthineers) was used for AI-based assessment of aortic calcifications and mean vertebral body densities. The calcification burden of the thoracic aorta was expressed as absolute volume (mm³). Vertebral bone density (BMD) was approximated using average density values of the T12 vertebral body expressed in Hounsfield units (HU).

AI-based coronary artery calcifications (CAC) were evaluated using the AVIEW v1.1.42.8-win (Coreline Soft), enabling the fully automated quantification of CAC across all four major coronary arteries on non-ECG gated CT scans [34]. To investigate the total thoracic vascular calcification (TTVC) burden of the patients, the aortic calcification volumes and the CAC volumes were summed, expressed in mm³.

**Fig. 2:**
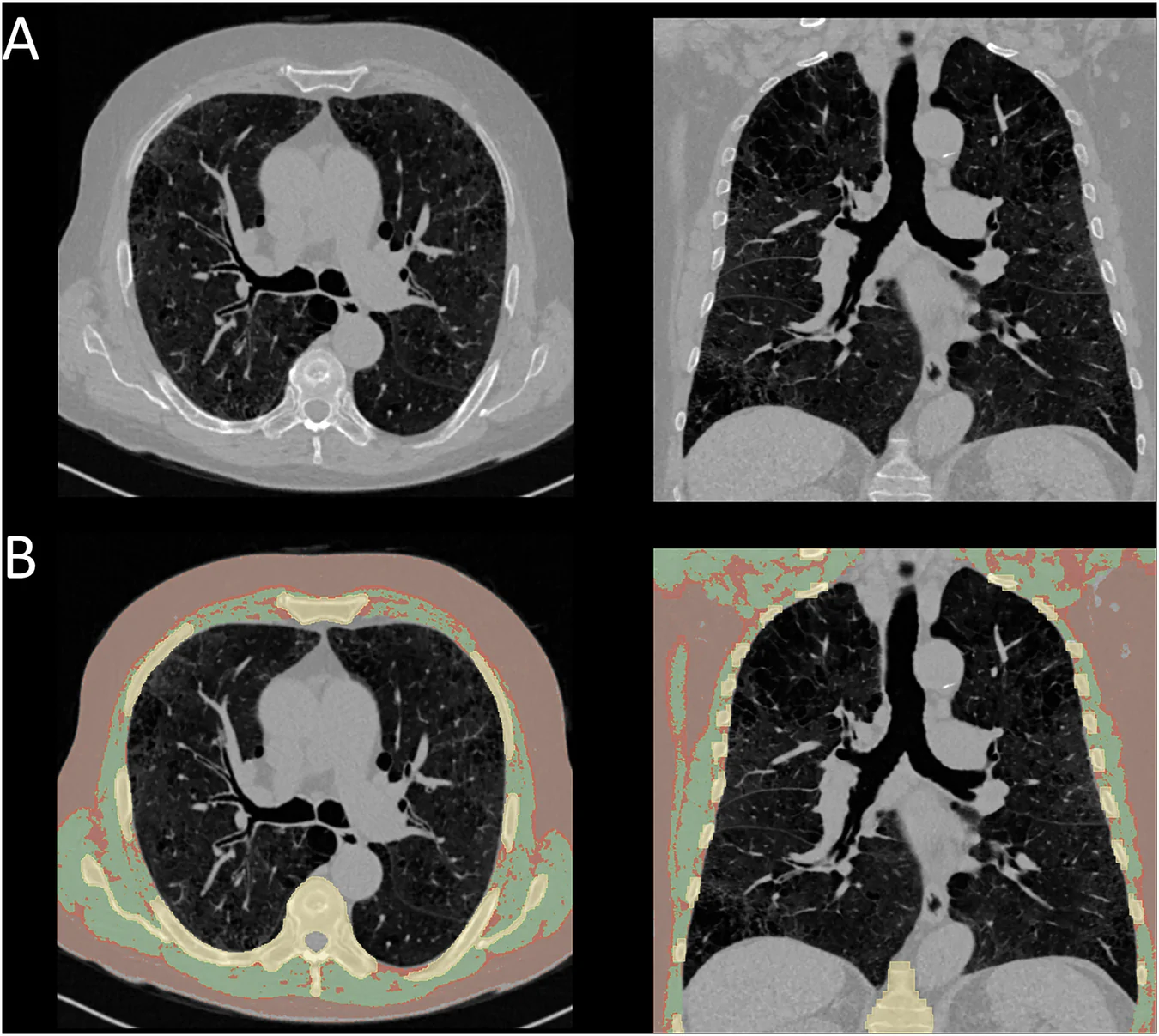
Artificial intelligence-based body composition analysis with the Body and Organ Analysis tool. **A** Axial low-dose chest CT scan and its coronal view. **B** Segmentation results of fully automated body composition analysis, including muscles (green), intermuscular adipose tissue (orange), and subcutaneous adipose tissue (brown)

For the more detailed body composition analysis of the patients, the “Body and Organ Analysis” tool (BOA) [35–37] was applied to the CT scans. With a fully automated detection of the thorax cavity, the tool provides volumetric measurements of the muscles, subcutaneous adipose tissue (SAT), visceral adipose tissue (VAT), and intermuscular adipose tissue (IMAT) (Fig. 2). For this study, the volume metrics were normalized to the patients’ heights, and the resulting indices were expressed in liter/m^2^.

### Statistical analysis

All analyses were performed in Python 3.12.7. Normal distribution of continuous variables was assessed using the Shapiro–Wilk test, supported by visual inspection of histograms and Q-Q plots. Categorical variables are summarized as counts and percentages, while continuous variables are summarized as median and interquartile ranges. For group comparisons, either the Mann–Whitney U-test or the Kruskal–Wallis test followed by Dunn’s post hoc test with Bonferroni correction was used. Because the analyses were hypothesis-driven and based on pre-specified variables, no global adjustment for multiple comparisons was applied. For categorical data, either the chi-square test or Fisher’s exact test was used. Depending on the skewness of the variables, either z-score normalization or log transformation and subsequent z-score normalization were applied. CT-based body composition measures were log-transformed, followed by a sex-stratified z-score normalization.

To explore associations with TTVC, univariate and multivariable linear regression analyses were performed. Model building was performed based on complete-case analysis; no imputations were used. Multicollinearity was assessed using variance inflation factors (VIF), with values below 5 deemed acceptable. Interaction models were employed following a stepwise manner to test the effect of distinct groups of covariates on the bone-vascular axis, including BMI, clinical covariates, metabolic covariates, lung function covariates, physical performance covariates, and age. The models were compared based on the log-likelihood ratio test, Akaike Information Criterion (AIC), and Bayesian Information Criterion (BIC), as well as adjusted R^2^ values. Statistical significance was defined as *p* < 0.05.

## Results

### Demographic characteristics

A total of 539 participants were categorized into normal nutritional status (*n* = 117), non-sarcopenic obesity (*n* = 317), sarcopenia (*n* = 55), and sarcopenic obesity (*n* = 50). Group comparison revealed significant differences in age, sex, weight, and BMI. Body weight ranged from a median of 55.0 kg for the sarcopenia group to 85.0 kg for the non-sarcopenic obesity group. Similarly, median BMI values ranged from 19.6 kg/m² (sarcopenia) to 28.8 kg/m² (non-sarcopenic obesity). Moreover, significant differences were found in pack-years, GOLD class, lung function parameters, physical performance parameters, as well as metabolic lab test values. T12 BMD was the highest in the sarcopenia group (139.0 HU (108.5, 170.0)) and lowest in the sarcopenic obesity group (117.0 HU (90.2, 140.0) (*p* < 0.01)). The TTVC also showed significant differences (*p* = 0.03), ranging from 427.0 mm³ (66.6, 1857.4) in the normal nutritional status group, to 1053.5 mm³ (168.0, 3071.8) in the sarcopenic obesity group (Table 1).

**Table 1:** Descriptive statistics according to conventional body phenotype groups

Variable	Data availability	All (*N* = 539)	Normal nutritional status (*N* = 117)	Non-sarcopenic obesity (*N* = 317)	Sarcopenia (*N* = 55)	Sarcopenic obesity (*N* = 50)
Age (years)	539 (100%)	67.0 (60.0, 72.0)	67.0 (58.0, 72.0)	66.0 (60.0, 71.0)	64.0 (57.5, 70.5)	70.0 (64.2, 74.0)
Sex (female)	539 (100%)	217 (40.3%)	39 (33.3%)	117 (36.9%)	37 (67.3%)	24 (48.0%)
Weight (kg)	539 (100%)	78.0 (68.0, 89.0)	71.0 (65.0, 79.0)	85.0 (77.0, 94.0)	55.0 (50.0, 60.0)	70.0 (63.0, 76.8)
BMI (kg/m²)	539 (100%)	26.3 (23.4, 29.6)	23.9 (22.3, 25.8)	28.8 (26.4, 31.6)	19.6 (18.3, 20.9)	23.1 (22.1, 24.0)
Pack-years	539 (100%)	38.0 (17.0, 63.1)	34.5 (9.0, 49.5)	41.7 (21.3, 68.8)	29.4 (11.7, 44.5)	32.4 (17.9, 61.2)
FEV1 (L)	539 (100%)	1.7 (1.2, 2.2)	1.8 (1.3, 2.2)	1.7 (1.3, 2.3)	1.2 (0.9, 1.8)	1.5 (0.9, 1.8)
FVC (L)	539 (100%)	3.1 (2.5, 3.8)	3.4 (2.7, 4.0)	3.2 (2.5, 3.8)	2.8 (2.2, 3.3)	2.9 (2.1, 3.5)
Tiffeneau index (%)	539 (100%)	54.1 (44.3, 63.7)	52.8 (42.7, 61.5)	55.1 (45.8, 65.8)	51.7 (38.5, 60.7)	49.1 (36.3, 58.2)
DLCO (% predicted)	533 (98.89%)	57.2 (42.6, 73.1) (*n* = 533)	60.0 (42.9, 76.9) (*n* = 115)	60.5 (46.8, 74.9) (*n* = 316)	46.2 (35.8, 59.0) (*n* = 54)	42.9 (31.2, 59.6) (*n* = 48)
GOLD (class 3–4)	539 (100%)	194 (36.0%)	39 (33.3%)	102 (32.2%)	29 (52.7%)	24 (48.0%)
TUG time (seconds)	534 (99.07%)	6.4 (5.4, 7.5) (*n* = 534)	6.0 (5.0, 7.0) (*n* = 117)	6.5 (5.5, 7.8) (*n* = 313)	6.4 (5.5, 7.0) (*n* = 55)	6.7 (5.8, 8.0) (*n* = 49)
6-MWD (m)	538 (99.81%)	453.0 (390.0, 516.0) (*n* = 538)	480.0 (417.0, 550.0) (*n* = 117)	450.0 (390.0, 510.0) (*n* = 317)	454.0 (390.0, 505.0) (*n* = 55)	425.0 (330.0, 463.0) (*n* = 49)
IPAQ (MET-min)	536 (99.44%)	2772.0 (1112.2, 6959.2) (*n* = 536)	4243.5 (1904.6, 8377.5) (*n* = 116)	2636.0 (1064.5, 6498.0) (*n* = 315)	3239.0 (1123.5, 7876.5) (*n* = 55)	1822.5 (618.8, 3902.2) (*n* = 50)
BODE index	538 (99.81%)	1.0 (0.0, 3.0) (*n* = 538)	1.0 (0.0, 2.0) (*n* = 117)	1.0 (0.0, 3.0) (*n* = 317)	3.0 (1.0, 4.0) (*n* = 55)	2.0 (1.0, 5.0) (*n* = 49)
T12 mean attenuation (HU)	539 (100%)	125.0 (98.0, 156.0)	134.0 (103.0, 169.0)	122.0 (93.0, 152.0)	139.0 (108.5, 170.0)	117.0 (90.2, 140.0)
TTVC volume (mm³)	539 (100%)	608.8 (123.7, 1825.0)	427.0 (66.6, 1857.4)	630.7 (160.2, 1803.2)	542.9 (55.5, 1142.1)	1053.5 (168.0, 3071.8)
Muscle index (L/m²)	539 (100%)	1.1 (0.9, 1.3)	1.2 (1.0, 1.4)	1.1 (1.0, 1.3)	1.0 (0.9, 1.1)	1.0 (0.8, 1.1)
SAT index (L/m²)	539 (100%)	1.0 (0.8, 1.4)	0.8 (0.6, 1.1)	1.3 (1.0, 1.6)	0.5 (0.3, 0.7)	0.9 (0.7, 1.1)
IMAT index (L/m²)	539 (100%)	0.4 (0.3, 0.5)	0.3 (0.3, 0.4)	0.5 (0.4, 0.6)	0.2 (0.1, 0.3)	0.4 (0.3, 0.4)

The results are expressed as median and interquartile ranges for continuous variables and as absolute numbers and percentages for categorical variables

*BMI* body mass index, *BODE* body mass index, airflow obstruction, dyspnea, and exercise capacity index, *DLCO* diffusing capacity of the lungs for carbon monoxide, *FVC* forced vital capacity, *HU* Hounsfield unit, *IMAT* intermuscular adipose tissue, *IPAQ* International Physical Activity Questionnaire, *SAT* subcutaneous adipose tissue, *TTVC* total thoracic vascular calcification, *TUG* timed up and go (test)

### Association of clinical parameters with TTVC

The association of the clinical variables with TTVC was investigated with linear regression analysis in a stepwise manner (Table 2). The first multivariable model (Model 1) included only clinical variables, Model 2 was adjusted for conventional body composition phenotypes, while Model 3 was adjusted for the CT-based body composition measures.

**Table 2:** Univariate and multivariable models for total thoracic calcification volume

	Univariate analysis		Model 1 (*n* = 473)		Model 2 (*n* = 473)		Model 3 (*n* = 473)	
Variable	Coefficient	*p*-value	Coefficient	*p*-value	Coefficient	*p*-value	Coefficient	*p*-value
Age (years)	0.60 [0.53–0.67]	**< 0.01**	0.54 [0.45–0.63]	**< 0.01**	0.55 [0.46–0.64]	**< 0.01**	0.54 [0.45–0.64]	**< 0.01**
BMI (kg/m²)	0.12 [0.03–0.20]	**< 0.01**	0.07 [−0.02 to 0.16]	0.11	0.1 [−0.02 to 0.22]	0.09	0.04 [−0.10 to 0.17]	0.59
Pack-years	0.19 [0.10–0.27]	**< 0.01**	0.15 [0.07–0.23]	**< 0.01**	0.16 [0.08–0.24]	**< 0.01**	0.15 [0.07–0.23]	**< 0.01**
HbA1C	0.26 [0.17–0.34]	**< 0.01**	0.08 [0.003–0.16]	**0.042**	0.08 [0.002–0.15]	**0.044**	0.08 [0.003–0.16]	**0.04**
FEV1 (L)^a^	−0.14 [−0.22 to −0.05]	**< 0.01**	ex.	ex.	ex.	ex.	ex.	ex.
FVC (L)^a^	−0.13 [−0.22 to −0.05]	**< 0.01**	ex.	ex.	ex.	ex.	ex.	ex.
Tiffeneau index (%)	−0.04 [−0.12 to 0.04]	0.35	−0.04 [−0.14 to 0.06]	0.47	−0.04 [−0.14 to 0.06]	0.40	−0.04 [−0.13 to 0.06]	0.47
GOLD class	−0.04 [−0.21 to 0.14]	0.69	−0.01 [−0.22 to 0.20]	0.89	−0.03 [−0.24 to 0.18]	0.79	−0.02 [−0.23 to 0.19]	0.86
TUG time (seconds)	0.21 [0.13–0.30]	**< 0.01**	0.02 [−0.07 to 0.11]	0.66	0.02 [−0.08 to 0.11]	0.72	0.02 [−0.07 to 0.12]	0.61
6-MWD (m)	−0.19 [−0.28 to −0.11]	**< 0.01**	−0.06 [−0.17 to 0.05]	0.32	−0.05 [−0.17 to 0.06]	0.34	−0.05 [−0.16 to 0.06]	0.40
Gait speed (m/s)^a^	−0.19 [−0.28 to −0.11]	**< 0.01**	ex.	ex.	ex.	ex.	ex.	ex.
BODE index^a^	0.05 [−0.03 to 0.14]	0.24	ex.	ex.	ex.	ex.	ex.	ex.
T12 mean attenuation (HU)	−0.27 [−0.35 to −0.19]	**< 0.01**	−0.02 [−0.11 to 0.06]	0.56	−0.03 [−0.11 to 0.05]	0.49	−0.02 [−0.11 to 0.06]	0.59
SAT index (L/m²)	0.14 [0.05–0.22]	**< 0.01**	–	–	–	–	−0.10 [−0.22 to 0.02]	0.11
IMAT index (L/m²)	0.22 [0.14–0.30]	**< 0.01**	–	–	–	–	0.14 [0.01–0.27]	**0.04**
Body composition (ref. normal)								
└ Non-sarcopenic Obesity	0.27 [0.06–0.48]	**0.01**	–	–	−0.01 [−0.22 to 0.20]	0.90	–	–
└ Sarcopenia	−0.03 [−0.35 to 0.29]	0.87	–	–	0.16 [−0.14 to 0.46]	0.29	–	–
└ Sarcopenic Obesity	0.37 [0.04–0.70]	**0.03**	–	–	−0.05 [−0.35 to 0.25]	0.75	–	–

Model 1: only clinical variables; Model 2: clinical variables + conventional body composition phenotypes; Model 3: clinical variables + CT-based body composition measures

*BMI* body mass index, *BODE* body mass index, airflow obstruction, dyspnea, and exercise capacity index, *HU* Hounsfield unit, *IMAT* intermuscular adipose tissue, *SAT* subcutaneous adipose tissue, *TTVC* total thoracic vascular calcification, *TUG* timed up and go (test)

Statistical significant results are highlighted

^a^ Variables excluded due to high correlation

After controlling for multicollinearity, 22 variables were retained in Model 1 (R² = 0.422, adjusted R² = 0.394). Age (β = 0.54, *p* < 0.01), pack-years (β = 0.15, *p* < 0.01), and HbA1C (β = 0.08, *p* = 0.042) reached statistical significance. The explained variance did not increase significantly (R² = 0.424, adjusted R² = 0.392; log-likelihood test *p* = 0.62) in Model 2, and none of the conventional body phenotypes were independently associated. Although in Model 3, adjusting for CT-based body composition measures, the IMAT index was identified as a significant variable (β = 0.14, *p* = 0.04) independent of age (*p* < 0.01), smoking (*p* < 0.01), and HbA1C (*p* = 0.04), while the explained variance increased to R² = 0.429, adjusted R² = 0.397; log-likelihood test *p* = 0.14.

### Association of T12 BMD with TTVC according to body phenotypes

#### T12 attenuation quantiles

**Fig. 3:**
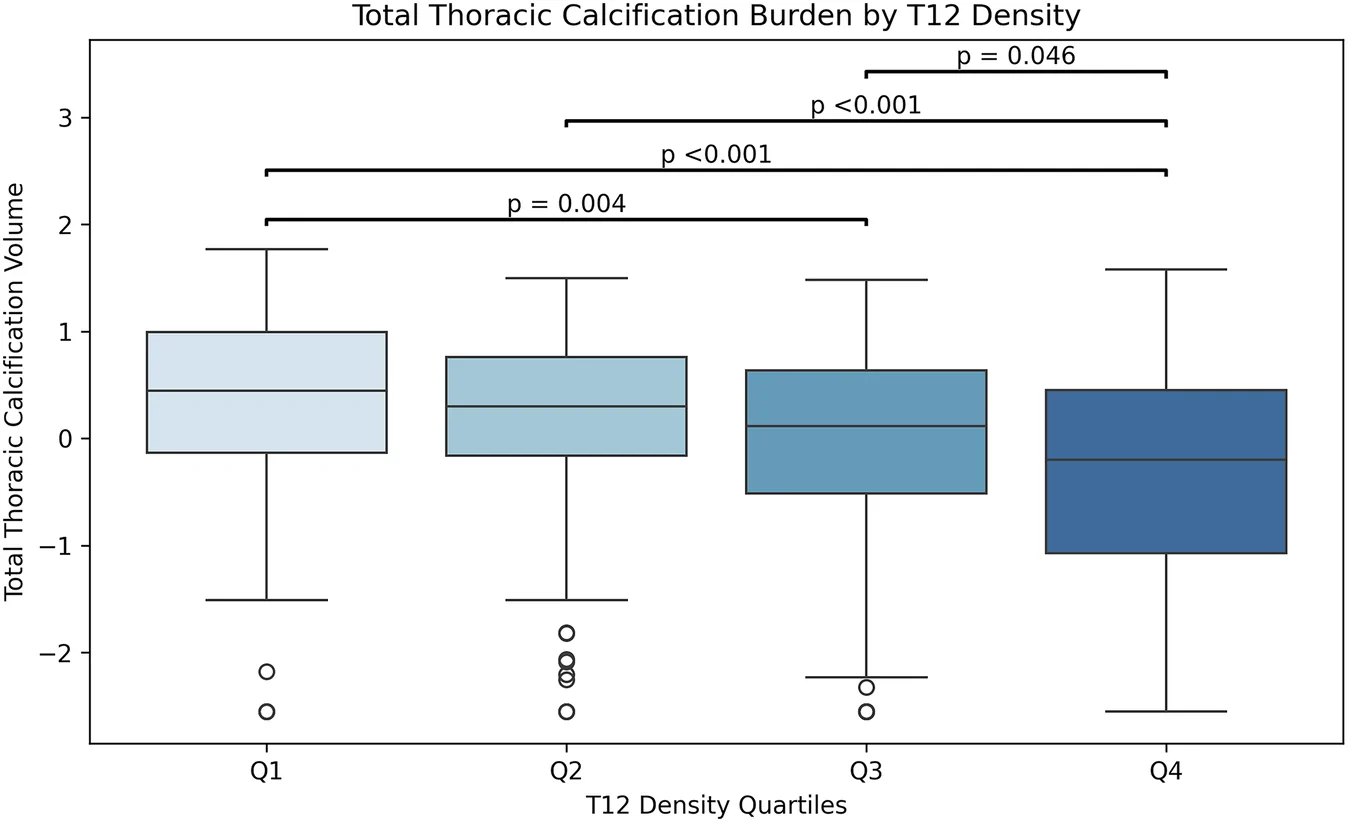
Association between T12 attenuation quartiles and total thoracic calcification volumes (TTVC). The boxplot shows a significant inverse trend in TTVC across increasing T12 attenuation quartiles

To explore the relationship between T12 BMD and TTVC, participants were stratified into BMD quartiles (Q1–4). The TTVC volume was the highest in Q1 and declined progressively across higher quartiles, with significantly higher TTVC volumes in Q1 compared to Q3 (*p* < 0.01), Q1 to Q4 (*p* < 0.01), Q2 to Q4 (*p* < 0.01), and Q3 to Q4 (*p* = 0.046), indicating an inverse association (Fig. 3).

#### The effect of body phenotypes

The negative association between T12 BMD and TTVC was consistent across all conventional body phenotypes, reaching statistical significance in the normal nutritional status phenotype (β = −0.38, *p* < 0.01), sarcopenia (β = −0.36, *p* < 0.01), and non-sarcopenic obesity phenotype (β = −0.24, *p* < 0.01). However, in the interaction model for TTVC, including T12 BMD and the body phenotypes, the phenotypes did not demonstrate a significant effect except the interaction term of T12 BMD × sarcopenic obesity phenotype (β = 0.41, *p* < 0.01), which lost significance after adjusting for other confounders.

### Difference between low-IMAT and high-IMAT patient groups

Based on the median IMAT index value, the patients were then divided into two groups. The high-IMAT group showed a significantly higher BMI (*p* < 0.01), SAT index (*p* < 0.01), pack-years (*p* < 0.01), and HbA1C (*p* < 0.01), together with a lower HDL (*p* < 0.01). They also showed worse functional performance with higher TUG time (*p* < 0.01), shorter 6-MWD (*p* < 0.01), and lower physical activity (IPAQ) (*p* = 0.03). Although the high-IMAT group had less GOLD 3–4 (*p* = 0.03) patients, together with higher Tiffeneau index (*p* < 0.01) and DLCO (*p* < 0.01), they reported more respiratory symptoms achieving a higher CAT score (*p* < 0.01), and mMRC grade (*p* < 0.01), which is also reflected in the distribution of ABCD classes (*p* < 0.01). In addition, T12 BMD was lower (*p* < 0.01) while TTVC was higher (*p* < 0.01) in the high-IMAT group. The detailed results are in Supplementary Table 1.

Fully adjusted linear regression models revealed different significant associations for the two groups (Table 3). For the high-IMAT group (R^2^ = 0.481, adjusted R^2^ = 0.418), age (*p* < 0.01), pack-years (*p* < 0.01), COPD disease duration (*p* = 0.04) and HbA1C (*p* = 0.04) reached significance, while for the low-IMAT patient group (R^2^ = 0.438, adjusted R^2^ = 0.372), SAT index (*p* = 0.046) and IMAT index (*p* < 0.01) were independently associated with TTVC besides age (*p* < 0.01) and pack-years (*p* = 0.01).

**Table 3:** Fully adjusted multivariable models stratified by IMAT index

	Low IMAT group (*n* = 233)		High IMAT group (*n* = 240)	
Variable	Coefficient^a^	*p*-value	Coefficient^a^	*p*-value
Age (years)	0.549 [0.41–0.689]	**< 0.01**	0.523 [0.389–0.657]	**< 0.01**
Sex (female)	−0.009 [−0.305 to 0.287]	0.95	−0.05 [−0.282 to 0.182]	0.67
BMI (kg/m²)	−0.036 [−0.275 to 0.203]	0.77	0.1 [−0.075 to 0.276]	0.26
COPD duration (years)	−0.104 [−0.22 to 0.011]	0.08	0.105 [0.005 to 0.206]	**0.04**
Smoking (pack-years)	0.144 [0.029–0.259]	**0.01**	0.151 [0.037 to 0.265]	**< 0.01**
Oral steroids	0.005 [−0.378 to 0.388]	0.98	0.217 [−0.159 to 0.594]	0.26
Any ICS	−0.138 [−0.38 to 0.104]	0.26	−0.087 [−0.292 to 0.117]	0.4
IL-6 (pg/mL)	0.052 [−0.064 to 0.169]	0.38	0.038 [−0.08 to 0.156]	0.53
IL-8 (pg/mL)	−0.025 [−0.149 to 0.1]	0.69	0.019 [−0.095 to 0.134]	0.74
TNF (pg/mL)	−0.023 [−0.141 to 0.095]	0.7	0.076 [−0.042 to 0.194]	0.21
HbA1C	0.029 [−0.093 to 0.15]	0.64	0.109 [0.007–0.21]	**0.04**
HDL (mg/dL)	−0.03 [−0.16 to 0.1]	0.65	−0.023 [−0.161 to 0.115]	0.74
Tiffeneau index (%)	−0.028 [−0.183 to 0.126]	0.72	−0.07 [−0.202 to 0.062]	0.30
DLCO (% predicted)	−0.051 [−0.204 to 0.102]	0.51	−0.025 [−0.162 to 0.112]	0.72
GOLD class (class 3–4)	0.126 [−0.183 to 0.435]	0.42	−0.181 [−0.477 to 0.116]	0.23
ABCD class	0.051 [−0.065 to 0.167]	0.39	−0.047 [−0.172 to 0.078]	0.46
TUG time (seconds)	0.091 [−0.079 to 0.261]	0.29	−0.009 [−0.121 to 0.102]	0.87
6-MWD (m)	0.025 [−0.15 to 0.199]	0.78	−0.066 [−0.212 to 0.08]	0.38
IPAQ (MET-min)	0.019 [−0.104 to 0.142]	0.76	−0.074 [−0.189 to 0.04]	0.20
CAT total score	0.109 [−0.024 to 0.242]	0.11	−0.018 [−0.146 to 0.111]	0.79
mMRC	−0.084 [−0.27 to 0.101]	0.37	0.079 [−0.081 to 0.239]	0.33
T12 mean attenuation (HU)	−0.092 [−0.219 to 0.035]	0.15	0.023 [−0.09 to 0.136]	0.69
Muscle index (L/m²)	0.076 [−0.046 to 0.197]	0.22	0.038 [−0.073 to 0.149]	0.50
SAT index (L/m²)	−0.213 [−0.422 to −0.004]	**0.046**	−0.07 [−0.247 to 0.106]	0.43
IMAT index (L/m²)	0.516 [0.209–0.823]	**< 0.01**	0.053 [−0.137 to 0.244]	0.58

*BMI* body mass index, *DLCO* diffusing capacity of the lungs for carbon monoxide, *HU* Hounsfield unit, *IMAT* intermuscular adipose tissue, *IPAQ* International Physical Activity Questionnaire, *SAT* subcutaneous adipose tissue, *TUG* timed up and go (test)

Statistical significant results are highlighted

^a^ 95% confidence intervals of the coefficients are reported

### Interaction models for the bone-vascular axis

In the baseline interaction model for TTVC, including T12 BMD, IMAT, and the interaction term T12 BMD × IMAT, all three variables reached statistical significance, suggesting that the inverse association between low bone density vs. high TTVC was amplified with increasing IMAT index. Across all models in the stepwise model building, IMAT remained independently associated, even after full adjustment, including age. BMI lost its significance once IMAT was added, confirming the superiority of IMAT. The interaction T12 BMD × IMAT was significant across the models until adding age, suggesting the moderation effect of IMAT on the bone-vascular axis is present but age-dependent. Smoking exposure, metabolic dysfunction, lung impairment, and physical frailty all incrementally added explanatory value, but age alone explained the largest share of variance by increasing the adjusted R^2^ by 0.17. Table 4 and Supplementary Table 2 summarize the results.

**Table 4:** The results of the stepwise interaction model building demonstrate the increase in explained variance and model fit with the log-likelihood ratio test

Model	Predictors added	Adj. R² (Δ)	AIC (Δ)	BIC (Δ)	χ²	*p*-value
Interaction model 0 (baseline)	Baseline interactions	0.109	1472.5	1489.6		
Interaction model 1	+ BMI	0.111 (0.002)	1472.2 (−0.3)	1493.7 (4.0)	2.3	0.13
Interaction model 2	+ Clinical covariates	0.141 (0.030)	1455.4 (−16.8)	1485.5 (−8.2)	20.8	< 0.01
Interaction model 3	+ Metabolic covariates	0.188 (0.047)	1282.6 (−172.9)	1341.2 (−144.3)	186.9	< 0.01
Interaction model 4	+ Lung function covariates	0.200 (0.012)	1261.1 (−21.5)	1357.0 (15.8)	39.5	< 0.01
Interaction model 5	+ Physical activity covariates	0.226 (0.026)	1233.4 (−27.7)	1345.7 (−11.3)	35.7	< 0.01
Interaction model 6 (fully adjusted)	+ Age	0.397 (0.170)	1116.7 (−116.7)	1233.2 (−112.6)	118.7	< 0.01

*AIC* Akaike Information Criterion, *BIC* Bayesian Information Criterion

## Discussion

Osteoporosis, obesity, sarcopenia, and arteriosclerosis are considered frequent comorbidities among COPD patients, with only limited knowledge available on their interaction. Our study on a multicentric cohort of 539 COPD patients investigated the role of body composition analysis in the context of the bone-vascular axis of osteoporosis. Although T12 BMD showed a consistent inverse association with TTVC across body phenotypes, the interaction model could not confirm a significant effect of phenotypes on the bone-vascular axis. The AI-based CT body composition showed a stronger contribution to the model; the IMAT index proved to be independently associated with TTVC even in the fully adjusted interaction model (β = 0.15, 95% CI 0.015–0.28, *p* = 0.029).

AI-based CT segmentation has shown promise in assessing body composition in COPD patients, demonstrating associations with disease severity and survival. Existing methods vary widely, with most studies limited to single-slice analysis [38]; however, emerging research suggests that multi-slice or volumetric analysis may be superior [39–42], paving the way for opportunistic screening of cardiovascular, metabolic, and skeletal comorbidities [43].

Previous studies investigated the different aspects of comorbidities in COPD patients with special focus on sarcopenia [13], osteoporosis [24], and coronary calcifications [44]; however, their interaction is still not fully understood. COPD patients show higher rates of osteoporosis; moreover, sarcopenia is also reported as a frequent comorbidity with varying prevalence (8–63%) [13, 24].

Surrogate markers of osteoporosis and sarcopenia, such as BMD and pectoral muscle area on chest CTs, were linked to physical function, disease severity, and mortality [45]. Moreover, BMD was associated with abdominal aortic calcifications [46], CAC, and vertebral fractures [47], and vice versa, a higher CAC was independently associated with lower BMD [48]. The bone-vascular axis was further analyzed in elderly SARS-CoV-2 patients, where the inverse association between aortic calcium score and L1 bone attenuation vanished after age adjustment [49]. In line with these findings, we observed higher TTVC in those with lower BMD; however, similar to prior reports, this relationship was not independent, underscoring the complex interplay of age and clinical factors in modulating the bone-vascular axis.

Previous studies have linked adiposity to CAC [50] and abdominal aortic calcifications [46]. Although we could not investigate VAT, we found that SAT and IMAT were associated with TTVC. Body composition phenotypes were also associated with cardiometabolic risk in a population study [51] reporting that both the “high adiposity–low muscle” and “high adiposity–high muscle” phenotypes showed adverse cardiometabolic indicators compared to the “low adiposity–high muscle” group, highlighting the critical role of adiposity regardless of muscle mass. In our study, BMD was inversely associated with vascular calcification across all phenotypes, although interaction models did not confirm a modifying effect.

The interplay of IMAT in COPD has emerged as a novel topic of interest. Jeon et al [52] reported lower normal-attenuation muscle area along with higher IMAT in COPD patients compared to the control group. Higher abdominal IMAT and lower psoas muscle volume were reported as associated with osteoporotic fractures independent of BMD in elderly men [53]. Increased intermuscular adiposity was also associated with greater all-cause mortality [38]. Lim et al [54] reported that the IMAT was the highest in sarcopenic obesity and non-sarcopenic obesity, in line with our results. Moreover, they highlighted that increased IMAT ratios were linked to adipose tissue inflammation and poorer functional outcomes. Consistent with these, we found the lowest lung function in the sarcopenic obesity and sarcopenia groups, suggesting muscle loss as a more important component than obesity in pulmonary impairment. In contrast, physical performance measures indicated obesity as the main determinant, as the sarcopenic group outperformed both non-sarcopenic obese and sarcopenic obese individuals.

Dividing the patients into low IMAT and high IMAT groups in our study identified an association of IMAT with obesity, an adverse metabolic profile, and functional impairment. The paradoxical finding of relatively preserved spirometry indices but greater symptom burden may point to IMAT reflecting systemic frailty and metabolic dysregulation rather than the severity of airflow limitation. The concomitant reduction in BMD and increase in TTVC in high-IMAT patients support that ectopic fat infiltration in muscle is linked to musculoskeletal aging and may contribute to the bone-vascular axis.

Moreover, stratified analyses revealed distinct variables independently associated with TTVC between low-IMAT and high-IMAT groups, implying that in patients with less muscle fat infiltration, body composition may have a more direct influence on vascular calcification, whereas in those with high IMAT, the calcification process is linked to accumulated metabolic injury and chronic disease burden.

Abdominal IMAT was previously associated with the prevalence of CAC [55]. Investigating thoracal IMAT, we observed comparable results: in stepwise interaction models, higher IMAT was consistently associated with greater TTVC, independent of BMI and other clinical covariates. Although age contributed the largest single improvement in model fit, IMAT still captured variance unexplained by age or BMI.

This study has noteworthy limitations. Its cross-sectional design limits conclusions regarding temporal relationships and causality, and the observed associations should be interpreted accordingly. Non-ECG-gated chest CT scans may lead to imprecise vascular calcification quantification, and scanner heterogeneity may introduce variability in imaging-derived measures. The lack of abdominal CT limited the analysis of VAT, the absence of a non-COPD control group limits interpretation of disease-specific effects, and selection bias remains a concern given the imaging-based subcohort. Additionally, confounders such as vitamin D levels, calcium metabolism, duration, and quantity of corticosteroid usage were not available, constraining the explanatory power of our multivariable models, although these factors may affect bone density and vascular calcification. Residual confounding cannot be excluded.

In conclusion, in this COPD cohort, IMAT, unlike BMI, was associated with an adverse body composition phenotype characterized by metabolic dysregulation, physical frailty, and musculoskeletal aging. These findings suggest that muscle fat infiltration may represent a marker of the bone-vascular axis in COPD, reflecting the link between impaired musculoskeletal health and vascular calcification. Future studies including non-COPD control groups and independent cohorts are needed to confirm these findings.

## Supplementary information

ELECTRONIC SUPPLEMENTARY MATERIAL

330_2026_12677_MOESM2_ESM

## Abbreviations

BMD: Bone mineral density
BODE index: Body mass index, airflow obstruction, dyspnea, and exercise capacity index
CAC: Coronary artery calcification
CAT: COPD assessment test
CT: Computed tomography
DLCO: Diffusing capacity for carbon monoxide
FFMI: Fat-free mass index
HU: Hounsfield units
ICS: Inhaled corticosteroids
IMAT: Intermuscular adipose tissue
IPAQ: International Physical Activity Questionnaire
MET: Metabolic Equivalent of Task
mMRC: Modified Medical Research Council dyspnea scale
SAT: Subcutaneous adipose tissue
TUG: Timed Up and Go Test
VAT: Visceral adipose tissue
