# Supplementary material for: Body composition’s effect on the bone–vascular axis of osteoporosis discovered in AI-based CT analysis of COPD patients
Source: Eur Radiol. 2026 Jul 3;36(10):7928–39. doi: 10.1007/s00330-026-12677-3 (PMC13574876; doi:10.1007/s00330-026-12677-3)
Supplement: Supplementary file 1 [file 330_2026_12677_MOESM1_ESM.pdf]

ELECTRONIC SUPPLEMENTARY MATERIAL

Body Composition's Effect on the Bone-Vascular Axis of Osteoporosis discovered in AI-based CT-Analysis of COPD Patients

Eur Radiol (2026) Budai BK; Wagner A; Havlicek O et al.

Supplementary Table 1. Differences between low- and high-IMAT index patient groups.

Variable	All (N=539)	Low IMAT (N=270)	High IMAT (N=269)	p-value
**Age (years)**	67.00 (60.00, 72.00)	66.00 (59.00, 71.00)	67.00 (61.00, 72.00)	0.15
**Sex (female)**	217 (40.3%)	111 (41.1%)	106 (39.4%)	0.75
**BMI (kg/m²)**	26.30 (23.40, 29.55)	23.90 (21.50, 26.00)	29.10 (26.50, 31.90)	**<0.01**
**COPD duration (years)**	8.00 (5.00, 12.00) (n=536)	8.00 (5.50, 12.00) (n=268)	8.00 (5.00, 12.50) (n=268)	0.97
**Smoking (pack-years)**	38.00 (17.00, 63.10)	32.75 (13.60, 54.10)	42.00 (22.00, 68.00)	**<0.01**
**Oral steroids**	48 (8.9%) (n=538)	26 (9.7%) (n=269)	22 (8.2%) (n=269)	0.65
**Any ICS**	314 (58.4%) (n=538)	160 (59.5%) (n=269)	154 (57.2%) (n=269)	0.66
**IL-6 (pg/ml)**	2.95 (0.51, 7.71) (n=499)	2.82 (0.41, 8.40) (n=241)	2.96 (0.55, 7.13) (n=258)	0.94
**IL-8 (pg/ml)**	8.13 (5.51, 11.66) (n=499)	7.72 (5.40, 10.81) (n=241)	8.73 (5.53, 12.37) (n=258)	0.16
**TNF (pg/ml)**	8.20 (5.04, 12.25) (n=499)	8.12 (5.10, 12.07) (n=241)	8.25 (5.00, 12.52) (n=258)	0.88
**HbA1C**	5.70 (5.40, 6.00) (n=528)	5.60 (5.40, 5.90) (n=262)	5.80 (5.50, 6.10) (n=266)	**<0.01**
**HDL (mg/dl)**	59.00 (49.00, 72.00) (n=522)	63.00 (51.75, 78.00) (n=260)	56.00 (48.00, 66.07) (n=262)	**<0.01**
**Tiffeneau index (%)**	54.12 (44.26, 63.74)	51.06 (40.57, 60.70)	56.61 (46.62, 66.05)	**<0.01**
**DLCO (% predicted)**	57.20 (42.60, 73.10) (n=533)	51.40 (39.10, 66.75) (n=267)	61.65 (47.45, 75.20) (n=266)	**<0.01**
**GOLD class (class 3-4)**	194 (36.0%)	110 (40.7%)	84 (31.2%)	**0.03**
**ABCD class**				**<0.01**
└ class A	75 (13.9%)	47 (17.4%)	28 (10.4%)	
└ class B	319 (59.2%)	139 (51.5%)	180 (66.9%)	
└ class C	6 (1.1%)	5 (1.9%)	1 (0.4%)	
└ class D	139 (25.8%)	79 (29.3%)	60 (22.3%)	
**TUG time (seconds)**	6.40 (5.40, 7.50) (n=534)	6.30 (5.20, 7.10) (n=270)	6.60 (5.70, 8.00) (n=264)	**<0.01**
**6-MWD (m)**	453.00 (390.00, 516.00) (n=538)	461.00 (398.50, 535.75) (n=270)	444.00 (378.50, 498.25) (n=268)	**<0.01**
**IPAQ (MET-min)**	2772.00 (1112.25, 6959.25) (n=536)	3339.00 (1200.00, 7533.00) (n=269)	2358.00 (942.00, 6439.50) (n=267)	**0.03**
**BODE index**	1.00 (0.00, 3.00) (n=538)	1.50 (0.25, 3.00) (n=270)	1.00 (0.00, 3.00) (n=268)	0.21
**CAT total score**	16.00 (12.00, 22.00)	16.00 (11.00, 21.00)	17.00 (13.00, 23.00)	**<0.01**
**mMRC**				**<0.01**
└ grade 0	62 (11.5%)	40 (14.8%)	22 (8.2%)	
└ grade 1	283 (52.5%)	150 (55.6%)	133 (49.4%)	
└ grade 2	135 (25.0%)	56 (20.7%)	79 (29.4%)	
└ grade 3-4	59 (10.9%)	24 (8.9%)	35 (13.0%)	
**T12 mean attenuation (HU)**	125.00 (98.00, 156.00)	130.50 (103.00, 159.75)	117.00 (92.00, 152.00)	**<0.01**
**TTVC volume (mm³)**	608.84 (123.66, 1824.99)	468.20 (70.05, 1597.87)	694.85 (183.87, 1938.62)	**<0.01**
**Muscle index (L/m²)**	1.11 (0.94, 1.32)	1.11 (0.95, 1.34)	1.11 (0.93, 1.31)	0.11
**SAT index (L/m²)**	1.05 (0.77, 1.39)	0.80 (0.62, 1.07)	1.30 (1.02, 1.63)	**<0.01**
**IMAT index (L/m²)**	0.43 (0.33, 0.54)	0.33 (0.25, 0.39)	0.55 (0.48, 0.64)	**<0.01**

The results are expressed as median and interquartile ranges for continuous variables and as absolute numbers and percentages for categorical variables. BMI: Body Mass Index; BODE: Body mass index, airflow Obstruction, Dyspnea, and Exercise capacity index; DLCO: Diffusing Capacity of the Lungs for Carbon Monoxide; FVC: Forced Vital Capacity; HU: Hounsfield Unit; IMAT: Intermuscular Adipose Tissue; IPAQ: International Physical Activity Questionnaire; SAT: Subcutaneous Adipose Tissue; TAT: Total Adipose Tissue; TTVC: Total Thoracic Vascular Calcification; TUG: Timed Up and Go (test)
